# A Distributed Approach for Collision Avoidance between Multirotor UAVs Following Planned Missions

**DOI:** 10.3390/s19102404

**Published:** 2019-05-26

**Authors:** Francisco Fabra, Willian Zamora, Julio Sangüesa, Carlos T. Calafate, Juan-Carlos Cano, Pietro Manzoni

**Affiliations:** 1 Department of Computer Engineering, Universitat Politècnica de València, 46022 Valencia, Spain; calafate@disca.upv.es (C.T.C.); jucano@disca.upv.es (J.-C.C.); pmanzoni@disca.upv.es (P.M.); 2 Faculty of Computer Science, Universidad Laica Eloy Alfaro de Manabí, Manta 130802, Ecuador; willian.zamora@live.uleam.edu.ec; 3 Universitary Center of Defense, General Military Academy, 50090 Zaragoza, Spain; jsanguesa@unizar.es

**Keywords:** unmanned aerial vehicle, sense and avoid, collision avoidance, ArduSim

## Abstract

As the number of potential applications for Unmanned Aerial Vehicles (UAVs) keeps rising steadily, the chances that these devices get close to each other during their flights also increases, causing concerns regarding potential collisions. This paper proposed the Mission Based Collision Avoidance Protocol (MBCAP), a novel UAV collision avoidance protocol applicable to all types of multicopters flying autonomously. It relies on wireless communications in order to detect nearby UAVs, and to negotiate the procedure to avoid any potential collision. Experimental and simulation results demonstrated the validity and effectiveness of the proposed solution, which typically introduces a small overhead in the range of 15 to 42 s for each risky situation successfully handled.

## 1. Introduction

The adoption of Unmanned Aerial Vehicles (UAVs) to perform a multitude of tasks is raising concerns about privacy, security, and flight safety [1], especially in urban environments where the consequences of any flight disruption are typically much more severe due to risks of citizen injuries. To address this issue, several efforts are taking place worldwide to make UAV flights safer. For instance, in Europe, U-space [2] is an initiative that aims at making UAV traffic management safer and more secure. In particular, U-space attempts to provide an appropriate interface with manned aviation and air traffic control so as to facilitate any kind of routine mission, in all classes of airspace, and even in congested environments like urban areas, so as to achieve the ambitious Single European Sky (SES) goal. The SESAR Joint Undertaking [3] was set up in order to manage this large scale effort, coordinating and concentrating all EU research and development activities onto air traffic management. This way, a wide range of drone missions that are currently being restricted will be possible thanks to a sustainable and robust European ecosystem that is globally interoperable.

Among the different areas where UAV flight safety is being considered, there is a particular area that has not yet been fully addressed: The development of sense and avoidance mechanisms to enable an UAV to become aware of its environment, allowing it to take evasive action if necessary [4]. In this paper we focused on this problem by proposing the Mission Based Collision Avoidance Protocol (MBCAP), a collision avoidance solution that relies on wireless communications between nearby UAVs performing planned missions. In particular, MBCAP-enabled UAVs will constantly broadcast their future positions, and whenever two UAVs determine that their flight trajectories overlap in time and space they will stop, and quickly negotiate and execute the process to safely go through the critical area. More specifically, we present MBCAP-e, an enhanced version of the original MBCAP-i protocol proposed in [5], that addresses many of the shortcomings detected in the earlier proposal. Among others, the most relevant improvements include:

- Collision risk distance changed from static (90 m) to dynamic by accounting for the current speed of the UAV;
- Beacon size was reduced, which reduces the channel occupancy;
- The safety distance between UAVs when one of them moves aside is also reduced;
- We have improved the optimal position of UAVs when moving aside so as to prevent collisions even when the flight trajectory is smoothed around waypoints.

Experimental results using real UAVs, along with large-scale simulation experiments, validate the effectiveness of our proposed protocol, and evidence the low overhead introduced both in terms of channel occupation and mission delays.

The remainder of this paper is organized as follows: In the next section we present the most relevant related works in this field. Then, in Section 3, we provide a quick overview of ArduSim, our simulation platform used to implement and test MBCAP. In Section 4, we present our MBCAP protocol, including all relevant technical details and improvements with regard to the initial version of the protocol proposed in [5]. Section 5 presents experimental results that validate our solution under different conditions, including both real flight data and simulation data, with discussion. Finally, Section 6 concludes the paper and refers to future works.

## 2. Related Works

Only recently has the topic of collision avoidance between UAVs gained more attention in the literature, and thus the number of works available remains limited.

Mahjri et al. [6] did a theoretical study of the characteristics that a collision avoidance protocol should have, describing its elements. In this work, the authors differentiate between two techniques for collision risk detection: Non-cooperative sensors, such as a proximity sensor [7] or a camera [8], and cooperative sensors, such as the dissemination of flight information to nearby UAVs, as occurs with Automatic Dependent Surveillance—Broadcast (ADS-B) [9] in the solution proposed by Liu and Foina [10]. In general, non-cooperative sensors can help avoid collisions with static objects, but they do not allow for a fast react enough to avoid collisions with moving objects, like other UAVs performing independent tasks. In these scenarios, cooperative sensors are more effective as the collision risk can be detected well in advance.

Jinwu et al. [11] defined a collision risk detection strategy based on space discretization. They assign a degree of danger to each location in the space following a probabilistic model that predicts the place an UAV will be in the future. This work focuses on UAVs moving very fast at a constant speed, and defines a vast protected area around the UAV, forcing other aircrafts to scatter over a wide area to avoid collisions. Furthermore, the authors did not explain the collision avoidance strategy used to change the direction of the UAVs during flight.

Lin et al. [12] presented an UAV collision avoidance solution which can achieve cluster situational awareness, autonomous formation control, and intelligent collaborative decision making. The main idea of their algorithm is to consider all swarm members as a whole, and control the internal and external parameters of the UAV swarm separately. Among the UAVs, a communication topology is set up. They used a consensus algorithm to maintain the formation and avoid collisions between UAVs. Moreover, they use the weight coefficient to set the priority for every UAV. Beyond single UAV control, an improved artificial potential field method is adopted to control swarm mobility. They improved the safety distance and the traditional artificial potential approach to make them more suitable for the UAV collision avoidance task. This way, even though UAVs approach obstacles at a high speed and with a small angle, they will still have enough time and space to change their flight direction. Authors validated the effectiveness of their cooperative obstacle avoidance algorithm using MATLAB alone.

Zhou et al. [13] presented a trajectory planning strategy for UAV collision avoidance. They proposed a varying cells strategy to integrate aerodynamic constraints into trajectory planning. They also adapted basic avoidance actions in the varying cells strategy to go through different cells, enabling more flexible avoidance maneuvers. Authors used Monte Carlo simulations to demonstrate that the proposed method satisfies aerodynamic constraints, while both the convergence and collision avoidance rates improved.

Kim and Ben-Othman [14] introduced a surveillance model for multi-domain Internet of Things (IoT) environments, which is supported by reinforced barriers with collision-avoidance using heterogeneous smart UAVs. Formally, they defined a problem whose goal was minimizing the movement of smart UAVs having as a condition that the collision-avoidance among UAVs was assured when flying between their initial positions and specific spots in a limited area.

Wang et al. [15] proposed an approach based on a 2D Laser Imaging Detection and Ranging (LIDAR) that offers a method to represent the objects in the environment in a compact manner, which was significantly more efficient in terms of both memory and computation in comparison with similar previous proposals. Their approach is also capable of classifying objects into categories such as static and dynamic, and tracking dynamic objects, as well as estimating their velocities with reasonable accuracy. The main problem of this proposal is that it was not designed for UAVs.

In [16], Ma improved a previous work by introducing collision and obstacle avoidance capabilities to target tracking. In particular, the author augmented the control input with a repulsion term that resolves collisions with other team members and nearby obstacles. Assuming that each UAV travels at a constant speed, a control component was added that adjusts the UAV’s heading angle to the opposite direction in relation to the UAV’s closest neighbors, and to obstacles that could provoke collisions. This repulsion term can also be expressed as a function of the relative bearing angles alone, making it possible to be estimated/measured by on-board vision sensors in the presence of communication losses. Regarding the communication topology tested, an all-to-all communication, a ring topology, and a cyclic pursuit topology were studied. The effectiveness of the proposal was demonstrated by only numerical simulation examples.

Chen and Lee [17] focused on proposing a novel and memory efficient deep network architecture named UAVNet for small UAVs to achieve obstacle detection in urban environments. The proposal shows that UAVNet can detect obstacles at a rate of 15 fps, meeting real-time application requirements.

To the best of our knowledge, no protocol has specifically addressed the issue of collision avoidance between multirotor UAVs from independent owners following planned missions, which is the scope of our current work.

## 3. ArduSim Simulator: An Overview

The MBCAP protocol has been developed using ArduSim, an accurate multi-UAV simulation platform developed by Fabra et al. [18] that has been freely released online [19] for the research community.

**Figure 1:**
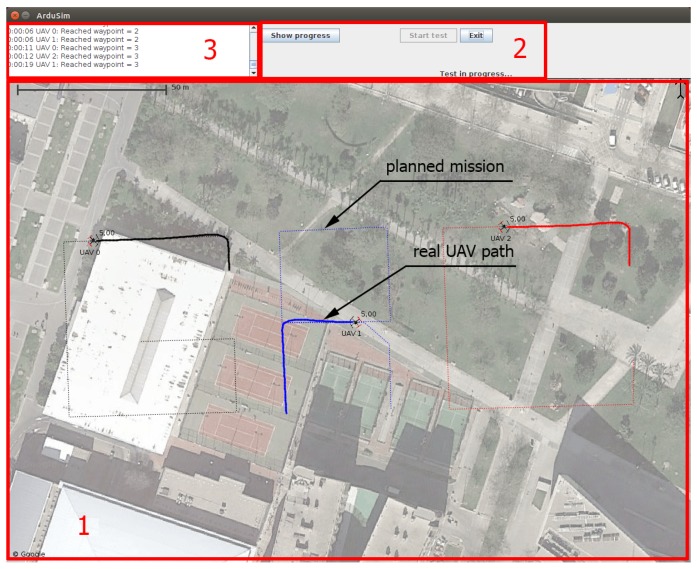
ArduSim main interface: Three Unmanned Aerial Vehicles (UAVs) following a mission.

Figure 1 shows the user interface of the ArduSim application. Most of it (area 1) is used to visualize how multicopters move during an experiment. The thin dotted line represents the mission the UAV has to follow, when applicable, and the thicker line represents the path it is actually following. On top, in the middle of the window (area 2), a few buttons allow the control of the experiment, and up on the left side (area 3), the log shows messages related to the progress of the simulation, and to the protocol under test.

Some of the features of ArduSim include:

- **Effortless protocol deployment on real UAVs**. Current open-source flight controllers use the Micro Aerial Vehicle Link (MAVLink) communications protocol [20] to communicate the UAV with an optional Ground Control System (GCS). ArduSim uses this protocol to fully control the behavior of the UAV while it is flying. The only requirements to deploy a protocol in a real multicopter are to attach a Raspberry Pi with a WiFi adapter (or a similar device capable of running Java), and to connect it to the telemetry port of the flight controller, following the instructions detailed on the ArduSim repository. ArduSim was designed to abstract the UAV control and communication layers to the developer, so that the same developed code works equally in simulation and in real UAVs, making the deployment straightforward.
- **Soft real-time simulation**. Simulations in ArduSim are performed in near real-time, which speeds up the debugging process while the protocol is implemented.
- **High scalability**. On a high-end computer (Intel Core i7-7700, 32 GB RAM, SSD hard drive), ArduSim is able to run up to 100 UAVs in near real-time, and up to 256 UAVs in soft real-time.
- **UAV-to-UAV communication simulation**. The communication among virtual UAVs is performed through virtual links based on 802.11a technology, using a model based on the results gathered from experiments with real multicopters. When the protocol is deployed in real multicopters, ArduSim automatically broadcasts User Datagram Protocol (UDP) datagrams, requiring a WiFi adapter connected to an Ad-hoc network.
- **Thorough control Application Programming Interface (API).** ArduSim provides a complete set of functions to perform the most common maneuvers during a flight: Take-off, start a mission, pause a mission, land, and so on.
- **Deployment through a PC Companion**. ArduSim can be run in three different roles: (i) Protocol testing on simulation, (ii) protocol deployment in a real multicopter, and (iii) as a PC Companion that helps to start and control the execution of the distributed protocol when deploying a real UAV swarm. Moreover, the PC Companion tool allows one to recover control over the UAVs in case the protocol does not behave as expected, thus avoiding any crash during the first tests with real UAVs.
- **Automatic collision detection**. Safety is a critical aim for any protocol. ArduSim informs the user if any collision happens during a simulation to help the researcher detect failures in the protocol design.
- **Comprehensive experiment data logging**. When the experiment ends, either in simulation or in a real multicopter, ArduSim stores, among others, the path followed by the UAV including coordinates, heading, speed, acceleration, distance to origin for each data recorded, as well as the same path in Google Earth [21], NS2 [22], and OMNeT++ [23] formats.

## 4. MBCAP Protocol

In this section we introduce MBCAP, which has been implemented with the ArduSim simulation platform. In particular, we detail MBCAP-e, an enhanced version of the original MBCAP-i protocol proposed in [5], that addresses many of the shortcomings detected in that earlier proposal.

### 4.1. Protocol Overview

MBCAP is applicable to UAVs following a planned mission in an autonomous manner, an issue not adequately addressed by the research community. To this aim, it relies on a cooperative sensing approach whereby multicopters broadcast their own location and predicted future locations. Upon receiving these data, receivers rely on it to decide if there is a collision risk (collision detection), and to avoid the collision if necessary (collision avoidance). The strategy is based on priorities, where an UAV has always a lower or higher priority than any other UAV it could meet during a flight. The high-priority UAV will be the first to resume its mission, and the low-priority UAV will wait the needed time to avoid the collision, only resuming its mission afterward.

Several technologies could be used to establish a communication link among the UAVs. ADS-B [9] could be a good solution, but it requires infrastructure, and uses proprietary technology and restricted frequencies. Our solution assumes the use of IEEE 802.11a wireless adapters operating in Ad-Hoc mode, an open and cheap solution already available in the market.

Regarding the architecture of the protocol, it comprises of three threads for each UAV, i.e., Beaconing, Listener, and CollisionDetector. The Beaconing thread periodically sends UDP broadcast datagrams with the current location of the multicopter, followed by a list of future predicted locations, including spatial and temporal coordinates. Such data are enough to detect collision risks with other UAVs. The Listener thread receives and stores the most up-to-date information received from other UAVs. Finally, the CollisionDetector thread periodically checks the gathered data and compares the future predicted locations with the ones advertised in its own beacon to decide if there is a collision risk with another UAV. If that is the case, it stops the multicopter and relies on the protocol to address the risky situation. The high-priority UAV resumes the mission when the other multicopter is ready to be overtaken. The low-priority UAV resumes the mission once the other one is in a safe location. Furthermore, if the low-priority UAV stands still in the path of the high-priority UAV, before giving way, it moves aside to let the UAV pass through its current location.

This protocol has been mainly designed to avoid collisions between two UAVs, as the probability of more than two UAVs performing planned missions to meet each other and all at once is very low. In case a third UAV detects a collision risk with any of the contending UAVs, it will stop and wait for the previous collision risk to be solved before applying the protocol.

The collision avoidance strategy is based on priorities at the time of deciding which UAV can go on with the mission. For this purpose, all the multicopters must have a unique identifier (ID) which enables us to establish an ordered relation among them. We used the unique ID value provided by ArduSim, defining the high-priority UAV as the one with the higher ID value. When ArduSim runs as a simulation environment, it provides random unique IDs for the virtual multicopters. However, when it runs in a real multicopter, it relies on the unique MAC address of the wireless adapter used for communications to generate a unique ID for the multicopter. This approach is good enough to analyze the performance of MBCAP for a general case of use. Later, for its deployment on commercial multicopters, the priority strategy should be analyzed more in depth, as some applications (e.g., border surveillance) may have higher priority than others (e.g., precision agriculture). In such cases, the priority of a UAV could defined at two levels: (i) Among UAVs running different applications, and (ii) among UAVs running the same application.

### 4.2. Finite State Machine

**Figure 2:**
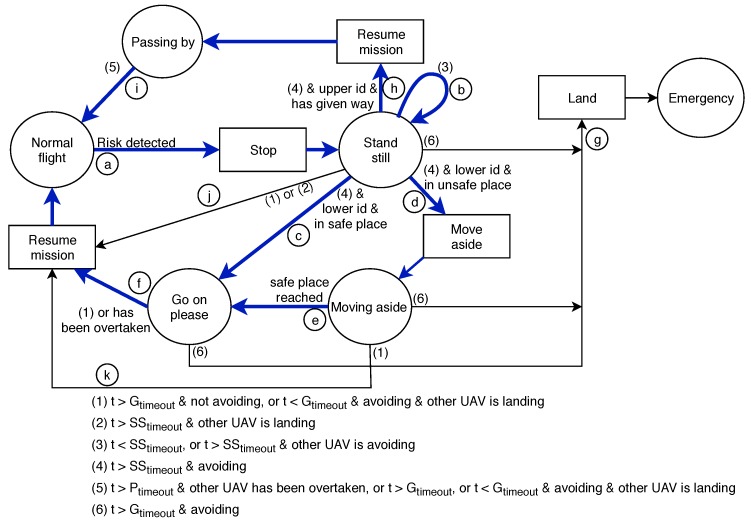
Mission Based Collision Avoidance Protocol-e (MBCAP-e) finite state machine.

In this section we detail the finite state machine that regulates the behavior of MBCAP-e, the new version of MBCAP, which is implemented in the CollisionDetector thread (see Figure 2). In the figure, the circles represent the machine states, the rectangles represent the commands sent to the flight controller to change the behavior of the UAV, and the arrows represent the transitions between states. The blue thick arrows are related to the most common scenario where only two UAVs are involved.

We will proceed to describe the most common situation addressed by MBCAP, where two UAVs meet and a collision risk is detected. Depending on the ID value, the UAVs can fall into any of the following cases:

1. LowerpriorityUAV. It starts in the Normalflight state. When a collision risk is detected, it needs a few seconds to stop in the air and enter in the Standstill state (transition *a*). Then, it will wait for a short time $SS_{timeout}$ (transition *b*) to ensure that the high-priority UAV has also reached the same state. When the other UAV informs that it is in the same state, it analyzes if it finds itself in the route the high-priority UAV was following. If not, it is safe to continue, and the UAV changes to the Goonplease state (transition *c*), allowing the other UAV to continue. On the contrary, it calculates where to move aside, and switches to the corresponding state (transition *d*), moving until it reaches the target location, and changing to the Goonplease state (transition *e*), as in the previous case. When the high-priority UAV moves beyond the area of conflict, the UAV resumes the mission (transition *f*) to exit the protocol, as the collision has been avoided.
2. HigherpriorityUAV. It also starts in the Normalflight state, and changes to the Standstill state (transition *a*) when a collision risk is detected. Then, it waits (transition *b*) for the same timeout until the lower priority UAV gives it way, resuming the mission (transition *h*), and changing to the Passingby state. Afterward, during the overtaking process, the high-priority UAV approaches the low-priority UAV. The overtaking ends when the former detects that the distance between them is increasing. Immediately, it informs the low-priority UAV that it can continue with the mission, and it simultaneously switches to the Normalflight state (transition *i*).

We have implemented MBCAP-e so as to be more resilient to unexpected situations, adding additional transitions. Thus, if an UAV is in a state different from Normalflight, and a global timeout elapses, it is because of these two cases: (i) If the UAV is not receiving messages from the UAV it is contending with, which means that there is no risk of collision, the mission is resumed (transitions *f*, *i*, *j*, *k*); otherwise, (ii) the UAV lands (emergency state) if the other UAV is close enough and the protocol has failed. The Passingby state is a special case where the UAV resumes the mission instead of landing, because the low-priority UAV has moved aside, if necessary, and there is no collision risk.

When a third UAV detects a risk of collision with one of the UAVs that are in the process of solving a collision situation, the protocol causes it to stop (transition *a*), and to wait in the Standstill state (transition *b*) until the previous risky situation is solved. Afterward, the protocol is applied between the two UAVs in risk of collision.

### 4.3. Beacon Content

MBCAP is a protocol where the decisions take into consideration the state information sent by the different UAVs using beacons. These beacons are periodically broadcasted using UDP datagrams.

**Figure 3:**
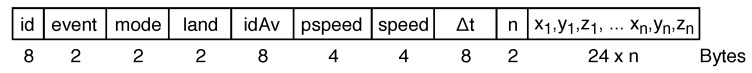
Periodic beacon content.

The beacon transmitted in MBCAP-e (see Figure 3) includes the following fields:

- id. Unique identifier of the sender UAV;
- event. Number of risky situations previously solved. The low-priority UAV resumes the mission when the high-priority UAV finishes the overtaking process and increases the value of this field;
- mode. Flight mode, equivalent to the current state in the finite state machine (see Figure 2);
- land. Whether the UAV started the landing phase. When an UAV reaches the end of the mission, it lands. MBCAP-e is not used while the UAV is landing because there are conditions under the landing procedure preventing it from being stopped;
- idAv. Identifier of the neighbor UAV with which the UAV is avoiding a collision, if applicable;
- pspeed. Planned ground speed for the mission (m/s);
- speed. Current ground speed (m/s);
- Δt. Time elapsed from the time the beacon information was generated until it has been transmitted; predicted future UAV locations are not recalculated for each beacon to avoid consuming excessive resources;
- *n*. Number of predicted future locations included in the beacon;
- Predictedlocationsarray. 3D Universal Transverse Mercator (UTM) coordinates for predicted future locations.

The array containing the future locations sent by each UAV in a beacon includes different information depending on its state. In particular, it will include the following information:

- Moving at low speeds (<1 m/s). Only the current location is broadcasted;
- Goonplease state. The current location and the location where the risk of collision was detected;
- Movingaside state. The current and the future locations towards the safe position the UAV is moving to;
- Standstill state. The current location and the set of waypoints not yet visited, conforming the information used to determine if the UAV should move aside to give way for a higher priority UAV, as detailed in Section 4.5;
- Normalflight state. The current location and future locations, used to detect a risk of collision with other UAVs.

### 4.4. Collision Risk Detection Strategy

A significant difference between an UAV manually controlled and an UAV following a mission is the fact that we can predict where the latter will be in the future, as it tries to follow a predefined path.

The strategy followed to detect a collision risk between two UAVs consists of predicting the future locations of the UAV given its current location, the waypoints it is moving towards (the remaining mission), and the current speed and acceleration. UAVs broadcast their future locations and periodically compare the received locations with their own predicted locations. If they match in both space and time, a collision risk is detected, and the UAVs stop. A match in space happens when the horizontal distance between the two UAV predicted locations is lower than 20 m, and the vertical distance is lower than 50 m. On the other hand, a match on time happens when the two predictions are within the same half second.

We already made a preliminary study on this strategy in [5], where the default configuration sends a beacon with an interval of 500 m/s between consecutive predicted locations. These locations are projected over the theoretical path the UAV must follow, and they are calculated considering the actual speed and acceleration provided by the flight controller. The acceleration is estimated to be constant throughout the flight time included in the prediction. In addition, since we observed that the calculated acceleration varies significantly, we decided to apply the following filter to the obtained value, with α=0.2 as determined in our previous study:

(1) $$a=\left\{\begin{array}{cc} 5 & \mathrm{if}a>5,\\ -5 & \mathrm{if}a<-5,\\ 0 & \mathrm{if}|*|a<0.1,\\ \alpha \cdot a_{i}-\left(1-\alpha \right)\cdot a_{i-1} & ,\alpha \in ]0,1]\mathrm{otherwise}. \end{array}\right.$$

By default, the predicted future locations are only updated once per second, but beacons are sent five times per second to make the protocol resilient to channel losses. The only field updated on a later beacon based on the same information is the time elapsed since the beacon was generated (field Δt, see Section 4.3).

MBCAP-e includes several improvements over the work in [5]. The previous version of the protocol (MBCAP-i) sent 50 predicted locations within each beacon, which corresponds to 25 s in the future. This configuration is prone to stop the UAVs too soon when their speeds differ significantly. In MBCAP-e, we just send the necessary amount of locations to detect a collision risk considering the current speed of the UAV (see Equation (2)).

(2) $$d=d_{GPS}+d_{brake}+d_{react}+d_{comm}$$

where:

$d_{GPS}=2.5\;m$
$d_{brake}=f\left(speed\right)$
$d_{react}=1\;s\times speed$
$d_{comm}=2\;s\times speed$

**Figure 4:**
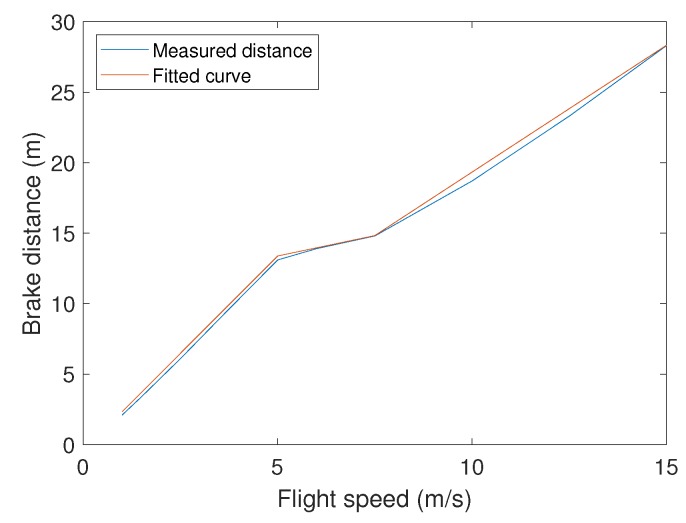
Measured brake distance vs. current flight speed.

We consider that there is a collision risk if the distance *d* between the UAV and the location where a collision risk is detected is lower than the combination of: (i) The GPS error ($d_{GPS}$), (ii) the distance required to brake ($d_{brake}$), being that the latter depends on the current UAV speed (see Figure 4), (iii) the distance traveled between two collision risk checks ($d_{react}$), and (iv) the distance traveled throughout a predefined time when considering that some messages can be lost during transmission ($d_{comm}$).

Given the safety distance, we calculated the total prediction time to be included in beacons as the safety distance divided by the current speed, and the number of locations to include in the beacon as the total prediction time divided by the time elapsed between two predicted locations (500 m/s in the default configuration). With the new configuration, the beacon only includes between 12 and 17 predicted locations depending on the speed, which represents a maximum of 9 s in the future, considerably lower than the original version of the protocol.

**Figure 5:**
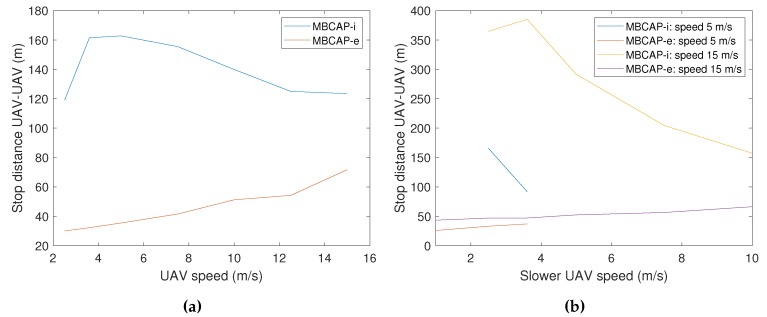
Distance between UAVs after stopping, in a face-to-face meeting. (**a**) UAVs flying at the same speed. (**b**) UAVs flying at different speeds.

**Figure 6:**
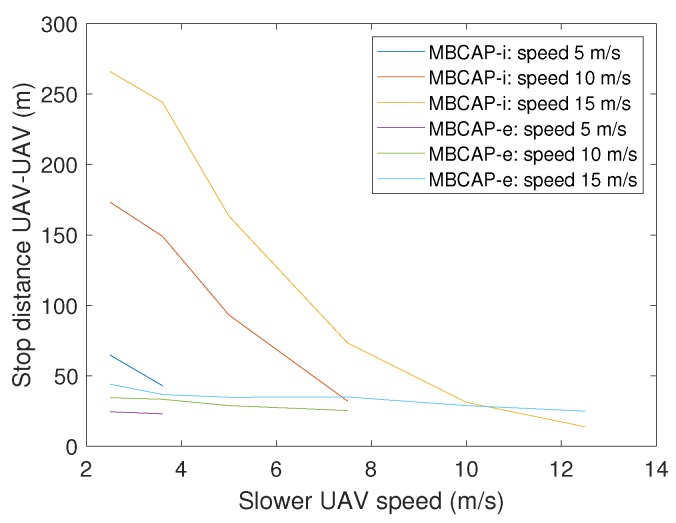
Distance between UAVs after stopping in a standard takeover, both flying at different speeds.

Figure 5a compares the distance between the UAVs when they stop due to a collision risk in the original (MBCAP-i), and the enhanced version of the protocol (MBCAP-e), both flying at the same speed. In the previous version, the UAVs stop too soon, causing the low-priority UAV to wait for a long time period until the high-priority UAV overtakes it. Furthermore, the distance between them increases as the speed goes down. On the other hand, with MBCAP-e, the distance is significantly lower, and it increases with the speed. Figure 5b and Figure 6 show similar results when the UAVs travel at different speeds. As stated before, the original version of the protocol is prone to stop the UAVs too soon when their speeds are quite different. With MBCAP-i, when the UAVs meet face-to-face, this distance could be up to 380 m, and when one of them overtakes the other from behind, it could be up to 260 m, making the process unnecessarily slow. With MBCAP-e, the distance becomes almost independent of speed differences and is significantly lower.

In order to enhance the performance of the protocol, we have introduced additional improvements regarding the information included in the beacon:

- **Prediction window**. As mentioned above, the number of future locations sent is reduced from 50 floats to a double number ranging from 12 to 17. This improvement reduces the size of the beacon from 640 bytes to 328–448 bytes, and reduces the CPU overhead while checking if there is a collision risk, thereby improving the overall quality of the prediction;
- **Beacon renewal**. If the protocol state of the UAV changes, we immediately update the predicted locations in the beacon;
- **Location accuracy**. The predicted locations were originally sent as UTM coordinates in float numbers, and now, in MBCAP-e, they are sent as double numbers instead, which increases the precision when detecting possible collision risks;
- **Braking awareness**. Now, only the current location of the UAV is sent if it is braking (a<−0.6 m/s^2^).

**Figure 7:**
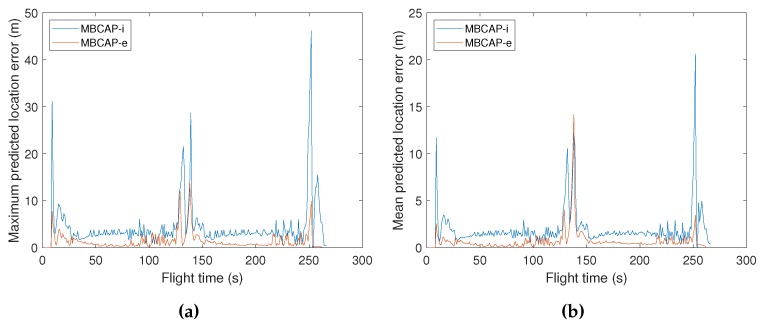
MBCAP-i vs. MBCAP-e: Predicted location error vs. flight time. (**a**) Maximum predicted location error. (**b**) Average predicted location error.

**Figure 8:**
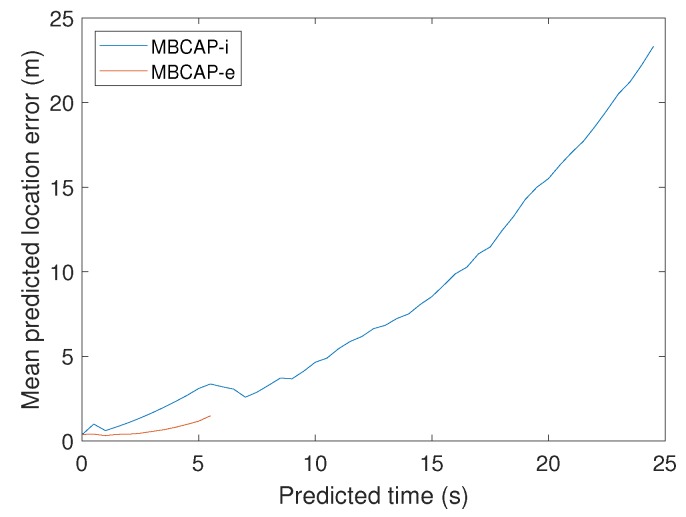
MBCAP-i vs. MBCAP-e: Average predicted location error for each of the predicted positions on the beacon.

All of these changes have improved the overall quality of the prediction. To check the accuracy improvement of the mechanism used to predict future locations, we performed experiments with a single virtual multicopter, measuring the distance between the predicted locations advertised in beacons and the actual UAV location at the predicted time. The UAV had a programmed speed of 15 m/s, and followed a route composed by two perpendicular segments, thereby representing very unfavourable conditions, since it is the maximum speed supported in mission-driven flights on common multicopters, and also because it represents a very pronounced turn. Figure 7a compares the maximum predicted location error for the original protocol and MBCAP-e, and Figure 7b shows the mean error. It can be observed that, when the UAV accelerates at the beginning of the experiment, while it takes the curve (second 140), and when it brakes for landing, the prediction error is higher. The error when flying at constant speeds remains mostly uniform. In all cases, the error in MBCAP-e is significantly lower than in the original version of the protocol, with a uniform maximum error of 1 m when the UAV traverses each segment of the mission at a constant speed. Figure 8 shows the average error for each of the predicted positions in the beacon along the whole experiment. We can see that there are much fewer locations being sent than before, and that the prediction quality increases, showing generally less error.

Additional enhancements have been included regarding the collision detection calculation. These changes, detailed below, improve the success rate at avoiding collisions, and reduce the time overhead introduced by the protocol (see Section 5):

- **Risk detection during landing disabled**. We disable the protocol when one of the involved UAVs is landing because some landing procedures cannot be stopped, making it impossible to apply the protocol;
- **Risk detection over time**. To detect if there is a collision risk we check if the predicted locations match in space and time. We only check if they match on time if the speed of both UAVs is greater than 1 m/s, and both beacons send more locations in addition to the current location of the UAV. This is a more conservative approach, as we assume that a stopped, or almost stopped UAV, will keep the location over time, and the approaching UAV will detect a risk when any of the future locations that it reports in the beacon matches in space with the location of the UAV that is standing still;
- **Short-term position status**. If the other UAV is in the Goonplease or the Standstill state, we only check if there is a collision risk with the current location of the UAV, ignoring the remaining locations included in the beacon. The first case includes the location where the UAV has detected the collision risk, and the second case includes a list of waypoints. None of these locations represent a position where the UAV will be on the short term, and so checking the existence of a possible collision risk at these locations would be inappropriate;
- **Fewer waypoints per beacon in the Standstill state**. In order to improve performance, we now include only the waypoints needed to report the path the UAV will follow for the next 400 m, a distance that is greater than the maximum distance between UAVs when they stop to avoid the collision;
- **Risk check timeout**. This new timeout works as follows: Once a collision risk has been avoided, we wait four seconds before attending any other UAV, informing that there is a collision risk with the current UAV (field idAv of the beacon). This approach solves a race condition in the protocol, due to its distributed nature, where the current UAV again detects a collision risk with the other UAV because the latter keeps signaling that the previous collision situation is still in the process of being avoided;
- **Global timeout set to 120 s**. If an UAV is in an state different from Normalflight for more than 120 s, it must resume the mission if possible (see Section 4.2), or land due to a deadlock condition associated to a protocol failure. This threshold is wide enough to consider the worst case, where the high-priority UAV (planned speed 1 m/s) could need 92 s to overtake the other UAV (planned speed 15 m/s). This situation could happen when the UAVs meet face-to-face.

### 4.5. Collision Avoidance Strategy

The global idea behind the collision avoidance strategy is to define a right of way based on priorities among the UAVs. For this purpose, we use a unique number (ID) based on the MAC address of the wireless adapter used for multicopters to communicate with each other. We use a random number provided by ArduSim when performing simulations. The UAV with a higher ID has right of way over the UAV with a lower ID.

**Figure 9:**
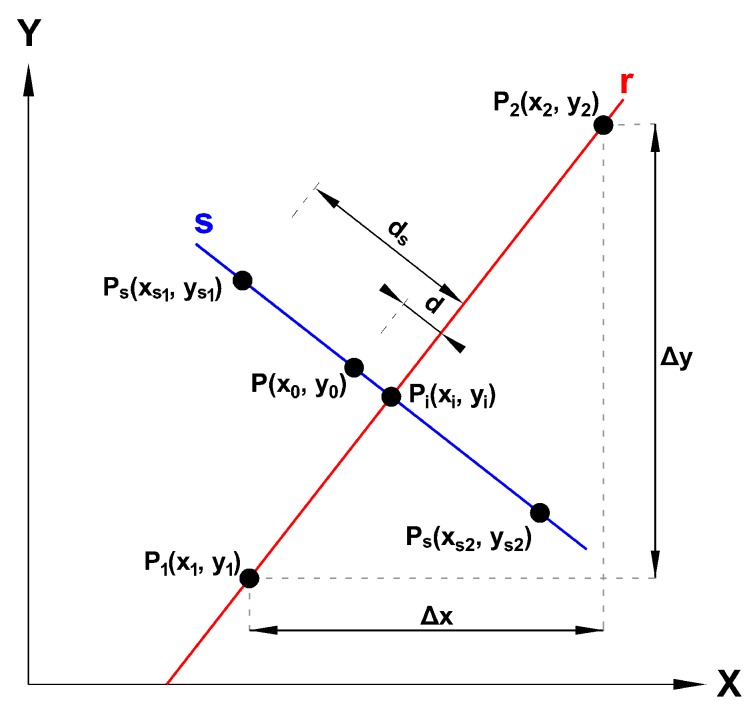
Safe location analysis.

Once a collision risk has been detected, both UAVs stop in the air and start the collision avoidance procedure. While they are in the Standstill state, they send their current location and a list with the next waypoints of the mission they have to follow. Figure 9 shows how the low-priority UAV determines, based on such beacon information, whether it should move aside to allow the other UAV to pass by, which happens when it stands on the path the high-priority UAV must follow. This is achieved by determining if the distance between its current location $P(x_{0},y_{0})$ and each of the mission segments *r* (delimited by $P_{1}\left(x_{1},y_{1}\right)$ and $P_{2}\left(x_{2},y_{2}\right)$), defined based on the locations advertised by the high-priority UAV, is higher than the threshold $d_{s}$. Notice that Δx and Δy refer to the relative increments on each axis according to the UTM coordinate system. Once the collision risk is detected, and to determine whether it is necessary for the lower-priority UAV to move to some specific location, it will follow these steps:

(1) Determining the intersection $P_{i}\left(x_{i},y_{i}\right)$ of line *r* (that contains the mission segment) with the perpendicular line *s* passing on point *P*:
  (3) $$P_{i}=\left(x_{i},y_{i}\right)=\left(\frac{y_{0}-y_{1}+\frac{\Delta x}{\Delta y}x_{0}+\frac{\Delta y}{\Delta x}x_{1}}{\frac{\Delta y}{\Delta x}+\frac{\Delta x}{\Delta y}},y_{1}+\frac{\Delta y}{\Delta x}\left(x_{i}-x_{1}\right)\right)$$
(2) If $P_{i}$ is within the mission segment, calculate the distance *d* between *P* and $P_{i}$.
(3) It is only necessary to move aside from this mission segment if the intersection $P_{i}$ is within the mission segment, and $d<d_{s}$. If $d_{12}$ refers to the distance between $P_{1}$ and $P_{2}$, in other words, it is the length of the segment, it is possible to calculate the coordinates for a safe location where to move ($P_{s}$) follows:
  (4) $$P_{s}=\left\{\begin{array}{cc} \left(x_{s_{1}},y_{s_{1}}\right)=(x_{i}-\frac{d_{s}}{d_{12}}|\Delta y|,y_{0}-\frac{\Delta x}{\Delta y}\left(x_{s}-x_{0}\right)) & \mathrm{if}\;x_{0}\leq x_{i},\\ \left(x_{s_{2}},y_{s_{2}}\right)=(x_{i}+\frac{d_{s}}{d_{12}}|\Delta y|,y_{0}-\frac{\Delta x}{\Delta y}\left(x_{s}-x_{0}\right)) & \mathrm{otherwise}. \end{array}\right.$$

If the low-priority UAV needs to move aside, it changes its location and gives way to the other UAV. Otherwise, it directly gives way. Then, the high-priority UAV starts the overtaking process, which finishes when (i) it goes beyond the collision risk region, and (ii) the distance to the low-priority UAV is increasing, and greater than 20 m.

**Figure 10:**
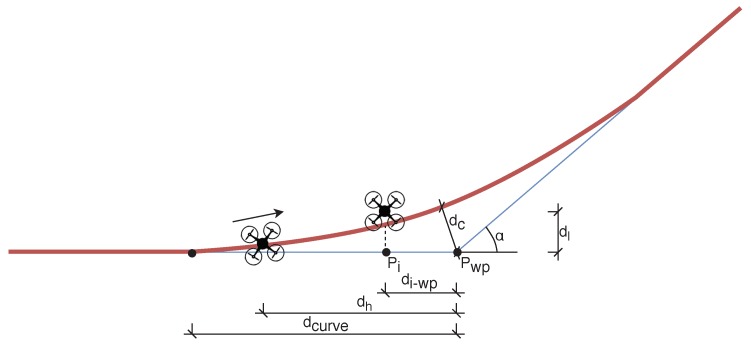
Safe location on curve analysis.

In order to improve the success ratio at avoiding collisions, and the overall performance of the protocol, several changes have been introduced in the collision avoidance strategy:

- **Reduced safety distance**. In the original version of MBCAP, when the low-priority UAV needs to move aside, it moves until the distance to the mission segment is of 20 m. We have rationalized this distance as the sum of the probable GPS error of both UAVs, an error margin due to detected errors of the flight controller while trying to take a curve at a high speed, and an additional error margin due to slight movements of the UAV while standing still:
  (5) $$d_{s}=2\times GPS_{error}+curve_{error}+position_{error}$$
  where:
  $$GPS_{error}=2.5\;m,\;curve_{error}=1.5\;m,\;position_{error}=1\;m;$$
- **Overtaking end behavior**. In MBCAP-e, the overtaking process does not finish until the high-priority UAV is at least 20 m (collision risk threshold) beyond the other UAV when the distance is increasing. As the safety distance is now lower than 20 m, without this requirement the UAV would immediately detect another collision risk with the low-priority UAV, and the protocol would be triggered again;
- **Waypoint behavior**. When the high-priority UAV is close to a waypoint (see the UAV with an arrow indicating its direction in Figure 10), it performs a curve to approach that waypoint, but without actually reaching it, and without stopping at all. If a collision risk is detected, both UAVs stop, but the previous approach to detect if the low-priority UAV is far enough from the path the other UAV is about to follow is not good enough, as there is an offset (dotted line) between the real path and the theoretical mission of the UAV. To consider this special case, we calculated the function that represents the maximum distance $d_{c}$ between the curve and the theoretical mission for different values of the planned speed and angle α between consecutive segments of the mission ($d_{c}=f\left(s,\alpha \right)$). If the high-priority UAV is close to a waypoint ($d_{h}<d_{curve}$), the angle between segments of the mission is reduced so as to trace a curve ($\alpha \in [-\frac{\pi }{2},\frac{\pi }{2}]$); then, if the low-priority UAV is in the inner side of the corner defined by the mission segments of the other UAV, and its distance $d_{l}$ to the mission segment is not safe ($d_{l}<d_{c}+d_{s}$), then we assume that there is a collision risk, and the UAV must move to the other side of the mission segment to guarantee safety.

## 5. Validation of the Proposed Solution

Having introduced the improved version of the MBCAP protocol, in this section we validate its correctness. To this end, we run ArduSim with different roles (see Section 3) to perform three different sets of experiments to analyze the behavior of the protocol under several circumstances: (i) UAVs approaching following straight trajectories under the most common scenarios, and with the presence of wind (ArduSim running as simulator), (ii) comparison of the results gathered in simulation with experiments using real multicopters (ArduSim running as simulator and also deployed on real UAVs), and (iii) analysis of the scalability and behavior of MBCAP when collision risks happen in scenarios with a large number of UAVs (ArduSim running as simulator).

### 5.1. Metrics

For our tests, we refer to the low-priority UAV with the number 1, and to the high-priority UAV with number 2, meaning that the UAV 1 is the one moving aside whenever necessary.

The main metric used to validate MBCAP-e is the success ratio at avoiding collisions between UAVs, and the second metric is the flight time overhead introduced by the protocol while it is being applied; in other words, it is the difference between the time needed by the UAV to finish the mission when it has solved one or more collision risks, and the time needed to accomplish the mission when no other UAVs are present.

### 5.2. Common Scenarios and Impact of the Wind

**Figure 11:**
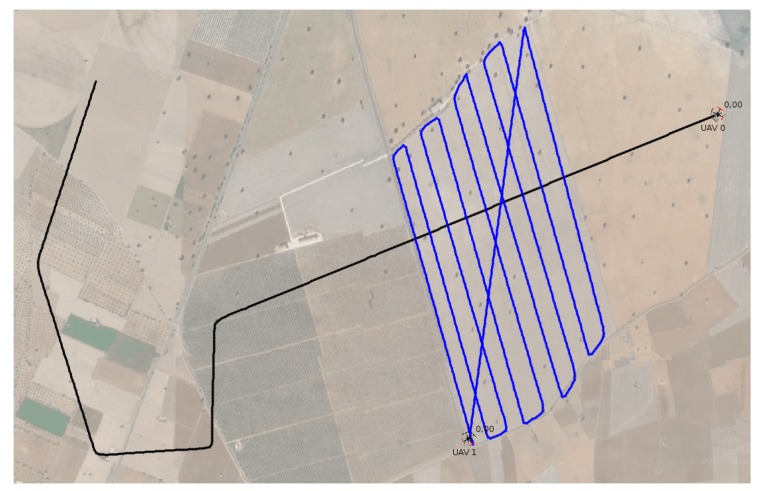
Collision avoided on a crop field (scenario 6).

The first set of experiments analyzes the most common scenarios where two UAVs approach each other following straight intersecting trajectories and considers different angles. Each of the following experiments was repeated three times, considering the worst result in all cases:

- Perpendicularcrossing (1). UAV 1 does not need to move aside;
- Standardtakeover (2). Both UAVs follow a similar trajectory, although UAV 2 is approaching UAV 1 from behind at a higher speed. UAV 1 must move aside so that UAV 2 can takeover;
- Face-to-facemeeting (3). This situation also requires UAV 1 to move aside in order to allow UAV 2 to pass without taking any risk;
- Angledtrajectories (4). UAV 1 does not need to move aside;
- Angledtrajectories,oppositedirections (5). Again, UAV 1 does not need to move aside;
- Supervisionofacropfield (6). It simulates a real situation where an UAV approaches another UAV that is supervising a crop field sized 1500×900 m, going through it in a zig-zag fashion (see Figure 11), following lanes separated by 100 m. The two UAVs meet with perpendicular trajectories when UAV 2 is on its third pass over the crop field, and UAV 1 does not need to move aside.

During experiments, the flight speed was set to 10 m/s, except for scenario (2) where UAV 1 was flying at a lower speed (5 m/s), so that UAV 2 was able to takeover. The collision was avoided in all cases and the time overhead (Δt) introduced by the protocol is shown in Table 1, comparing the original version of the protocol with MBCAP-e. In general, we found that the flight time overhead introduced by MBCAP-i for UAV 1 ranged between 35 and 56 s, and for UAV 2 it ranged from 18 to 29 s. On the other hand, MBCAP-e introduced an overhead of 27 to 42 s, and 15 to 24 s, respectively. The results show an overall gain for MBCAP-e in terms of flight time overhead, specially for UAV 1 (lower priority); such an improvement is mainly associated with having UAVs stop at a shorter distance between them.

We also tested if the presence of wind affects the correctness and performance of the protocol. Table 2 shows the results when introducing constant deterministic wind at a high speed (20 m/s) in scenarios (1) and (2). We observed that the flight time increased with headwind, as the UAV was no longer able to keep a ground speed of 10 m/s, and it had no significant variation when the UAV flew at 5 m/s, or if the sidewind or tailwind were present. Moreover, the flight time overhead introduced by MBCAP-i and MBCAP-e was not significantly affected by the wind speed.

Overall, we found that both versions of the protocol avoided the collision in all cases, with the flight time overhead introduced by MBCAP-e being significantly lower than for the previous version.

### 5.3. Simulation vs. Real Testbed for Common Scenarios

**Figure 12:**
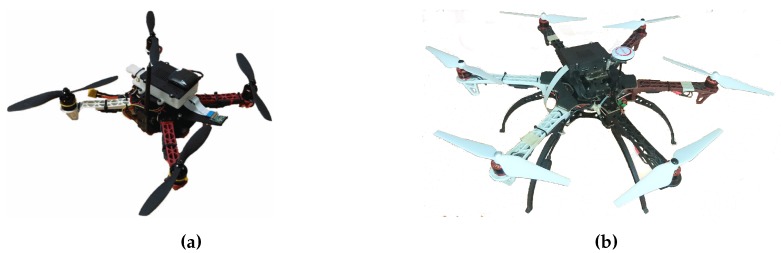
Multicopters used in real testbed. (**a**) Quadcopter, and (**b**) hexacopter.

**Figure 13:**
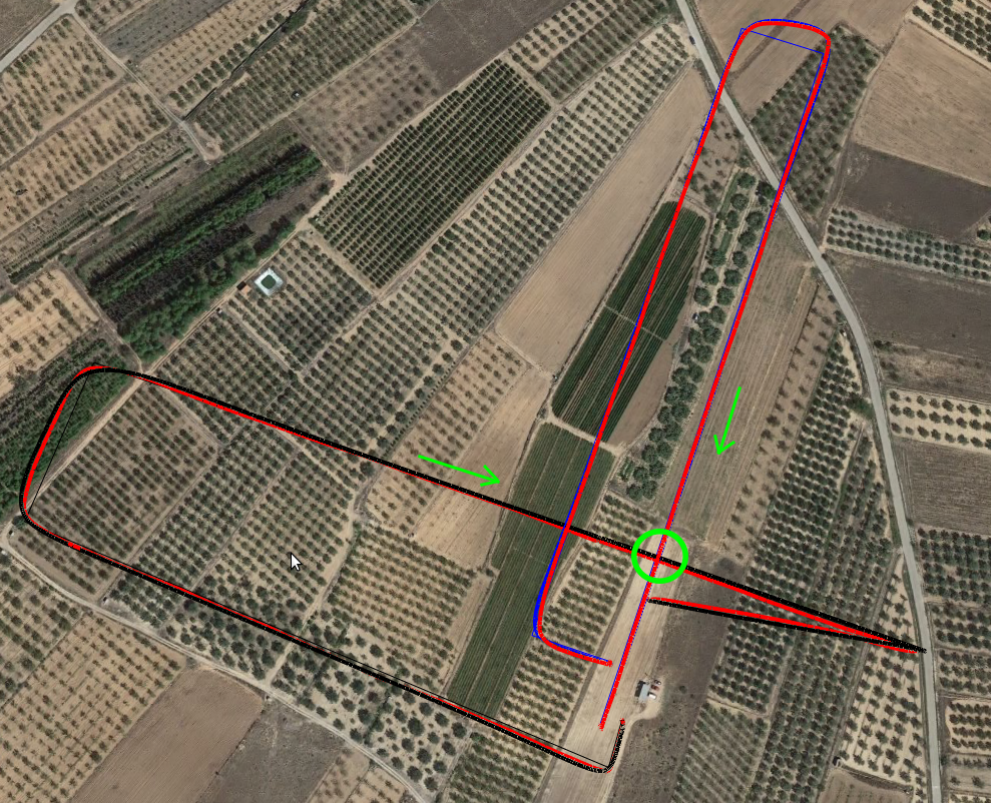
Simulation vs. real experiment in a perpendicular crossing (scenario (1)).

The second set of experiments analyzes the effectiveness and performance of MBCAP-e deployed on real multicopters. To this end, we deployed ArduSim with MBCAP-e in a GRCQuad quadcopter from Quaternium [24] (see Figure 12a), and in a customized hexacopter (see Figure 12b), both capable of running ArduSim with the multicopter role in a Raspberry Pi 3B+ attached to them, and connected to the telemetry port of the flight controller through a serial port link (detailed instructions are available in the ArduSim repository [19]). Experiments were performed for scenarios 1 to 5 from the previous section with similar missions, and then they were repeated without using MBCAP-e in order to measure the flight time overhead. In the experiments, the hexacopter had higher priority than the quadcopter. Finally, the experiments with and without MBCAP-e were repeated in simulation to compare both results. As an example, Figure 13 depicts a Google Earth 3D view that shows the path followed by the real multicopters with a red line, the path of the virtual high-priority UAV with a blue line, and the route of the virtual low-priority UAV with a black line. The green arrows indicate the direction the UAVs were moving towards before detecting the collision risk, also marked with a green circle. We observed that the paths followed in simulation and in real experiments were quite similar.

The collisions were avoided in all cases, both in simulation and in real experiments. Table 3 shows that, in general, the flight time overhead remains similar in both environments, with the exception of scenarios (1) and (5), where the presence of a gusty wind slightly increased the time necessary to complete the process. A video showing these experiments is also available online (https://youtu.be/xHnMuMOd9C0).

### 5.4. Scalability Analysis

In the previous sections we confirmed that MBCAP-e behaves better than its previous version. The protocol always avoids collisions whenever two UAVs meet in the air following a straight line from different directions, and the flight time overhead is bounded and low enough considering the battery capacity of current multicopters. Moreover, we showed that MBCAP can easily be deployed on real multicopters thanks to ArduSim’s capabilities. In this section, we analyze how the protocol behaves when the risk of collision increases, and the UAVs trace curves along their path, by simulating a large number of UAVs in a bounded area. A video that summarizes some of these experiments is also available online (https://youtu.be/bEdcsPX1hXY).

#### 5.4.1. Experimental Setup

MBCAP was tested on a squared area of 5×5 km, deploying 25, 50, 75, and 100 UAVs on four different scenarios. Each scenario consists of a new random deployment location for each UAV, and each UAV is assigned a new random mission based on the Gauss–Markov Mobility pattern [25] included in OMNeT++ [23]. Each experiment was repeated three times, taking the mean value. Moreover, the flight time and the traveled distance were measured with MBCAP-i, MBCAP-e, and without applying the protocol at all, in order to determine its overhead and performance. When the protocol is not used, the mean flight time was of 1 h and 4 min, the mean traveled distance per UAV was of 36.9 km, and the mean number of collisions was of 6.5, 16.5, 45.5, and 84.25 when deploying 25, 50, 75, and 100 UAVs, respectively. Furthermore, we found that the selected scenario does not significantly affect the experimental results.

**Figure 14:**
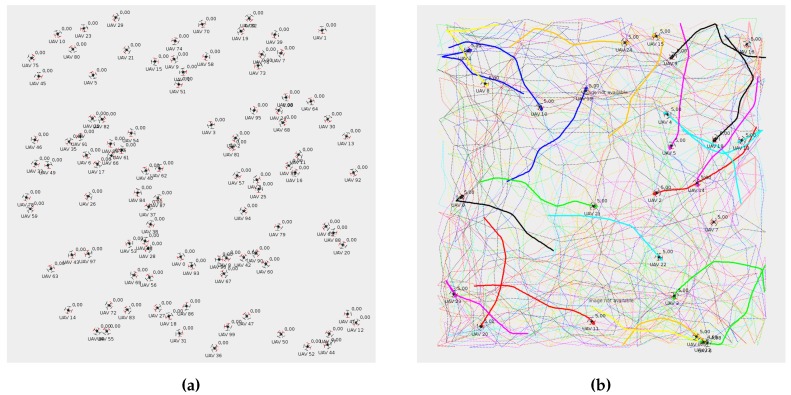
Experiment setup in an area of 5×5 km. (**a**) 100 UAVs randomly deployed. (**b**) 25 random Gauss–Markov missions.

We used Algorithm 1 to get a random location and heading for each UAV included in our experiments. Initially, all the UAVs were randomly located inside the target area and then, if needed, their initial location was adjusted so that the distance between them was greater than or equal to the minimum distance specified for the experiment (100 m). The minimum distance between UAVs had an upper limit to assure that they could fit inside the area ($d_{min}<\Delta x/\sqrt{n}$, and $d_{min}<\Delta y/\sqrt{n}$). Figure 14a shows the results gathered for a scenario with 100 UAVs randomly deployed.

**Algorithm 1:**RANLOC Returns a Random Initial Location and a Random Heading for *n* UAVs.

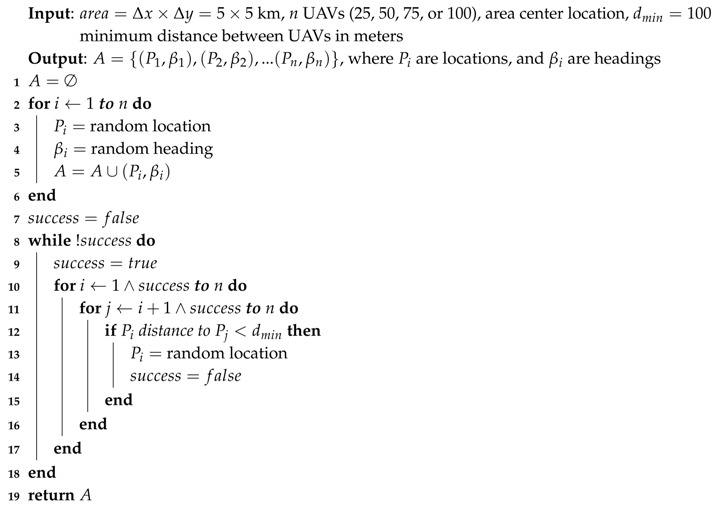



**Figure 15:**
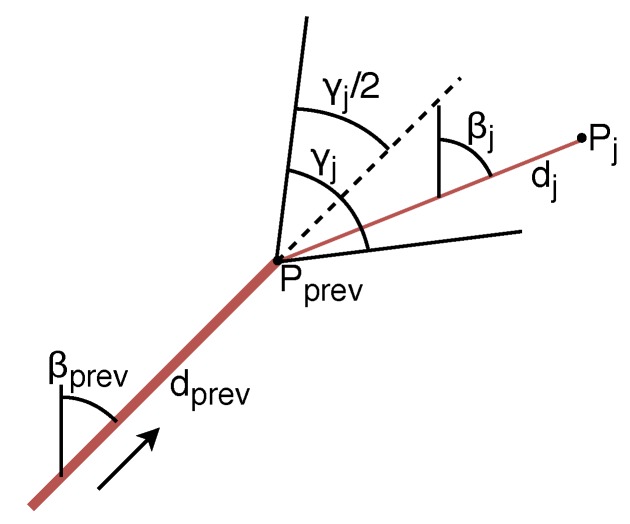
Gauss–Markov mobility model calculations.

The main objective of this set of experiments was to force the UAVs to meet several times in the air, not only when they were flying following a straight line, but also when they were performing a curve trajectory close to a waypoint. To this end, we designed long experiments where the UAVs changed their direction along almost one fourth of the mission length so as to create a highly unfavorable scenario. We used Algorithm 2 (see Figure 15) to get all the waypoints of the mission, starting from the initial location previously calculated. The length of each segment of the mission was randomly obtained in a range that varied from 250 to 500 m. Moreover, the maximum length should be lower than half the side of the area where UAVs were deployed so as to guarantee that the algorithm can go on. Global parameter α represents the linearity of the path followed by the UAVs, varying from 0 (Brownian movement) to 1 (linear motion). We set α to 0.75, which makes the mission significantly linear (see Figure 14b), changing to a Brownian movement when the UAV was too close to the limits of the area, with the aim of allowing it to bounce inward. Finally, we set the number of the mission’s waypoints to 100, which is equivalent to 99 segments having a mean length of 375 m.

**Algorithm 2:**RanMission Gets a Random Mission for *n* UAVs.

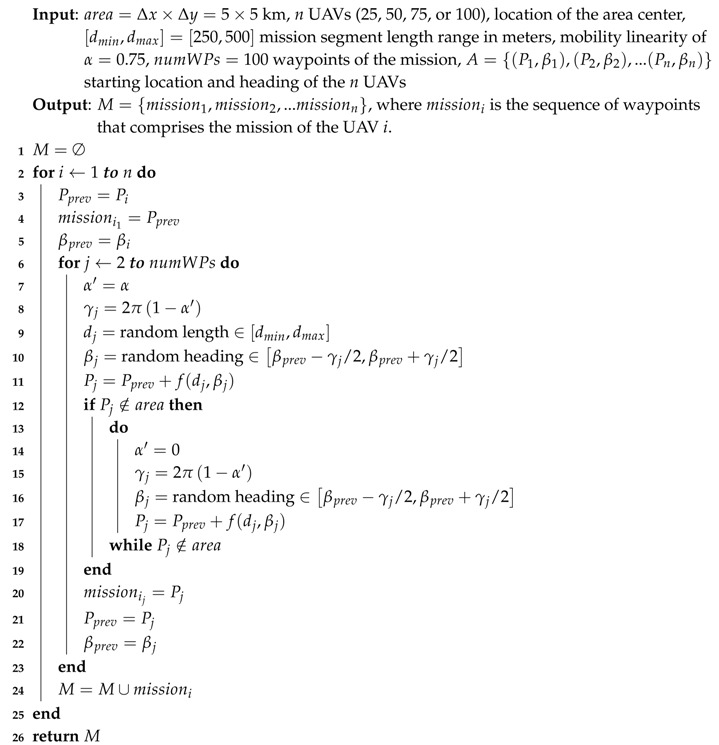



#### 5.4.2. Global Results

Table 4 compares the results gathered with MBCAP-i and with MBCAP-e, when varying the number of UAVs in the area. We show the mean value for all the experiments, finding that MBCAP-e significantly outperformed MBCAP-i. We now proceed to analyze the performance metrics included in Table 4.

**Figure 16:**
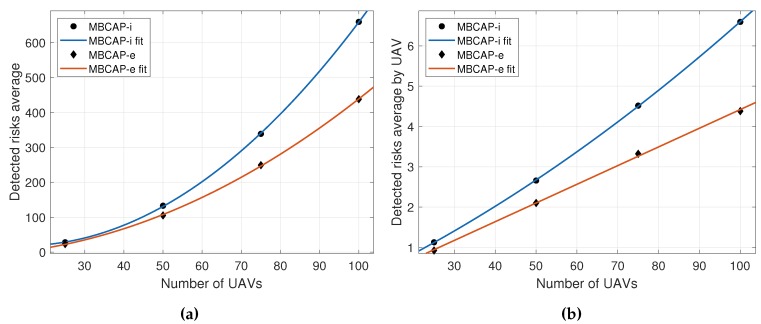
MBCAP-i vs. MBCAP-e: Average risks detected during an experiment. (**a**) Total risks vs. number of UAVs: $O(n^{2})$. (**b**) Risks by UAV vs. number of UAVs: O(n).

**Figure 17:**
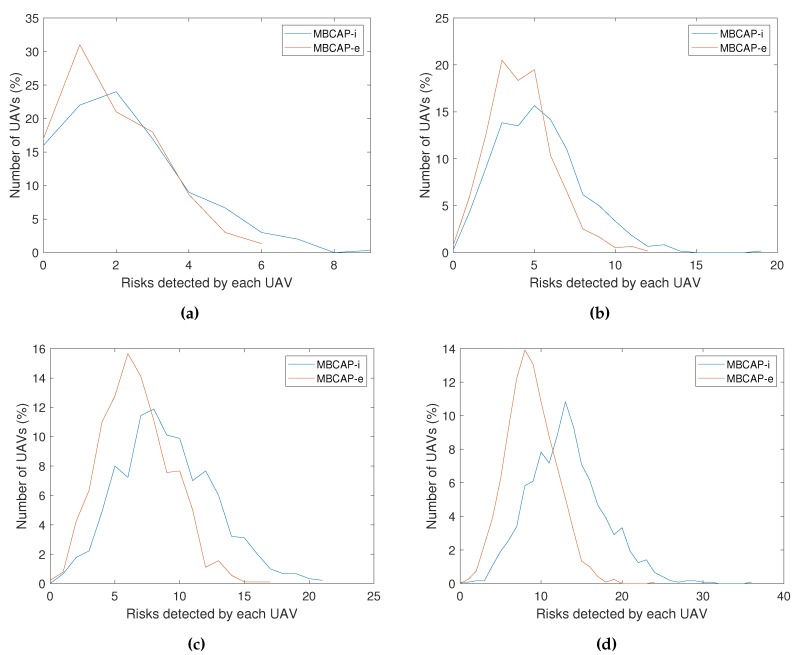
MBCAP-i vs. MBCAP-e: Distribution of UAVs given the risks detected by each one. (**a**) Experiment with 25 UAVs. (**b**) Experiment with 50 UAVs. (**c**) Experiment with 75 UAVs. (**d**) Experiment with 100 UAVs.

- **Collisions expected**. Represents the mean number of collisions detected between UAVs in a single experiment, when the protocol was not in use. This value allows us to determine the success ratio of the protocol at detecting and avoiding collisions;
- **Risks detected**. Represents the mean number of times the collision-avoidance protocol was enforced throughout the experiment. Figure 16a shows that the mean number of risky situations detected along an experiment increased with the number of UAVs present in the area as $O(n^{2})$, and it also showed the precision increment in the collision detection strategy that was achieved with MBCAP-e, where the latter was able to prevent UAVs from stopping unnecessarily in many situations. On the other hand, Figure 16b shows that the mean number of dangerous situations detected by an UAV in a single experiment increased with the number of UAVs as O(n). Finally, Table 4 and Figure 17 show that, in general, the UAVs detected less risky situations when adopting MBCAP-e, for any number of UAVs, e.g., 66.4% for 100 UAVs;
- **Soft collisions (d<5m)**. Mean number of possible collisions taking place during an experiment. We considered that a simulated collision happened when two UAVs was located at a distance lower than 5 m, meaning that a security cylinder of 5 m radius around each UAV. The typical GPS error on multicopters was 2.5 m, and so we considered it a very unfavorable situation, occurring in those cases where the GPS error bias of both UAVs was exactly the opposite. MBCAP-e highly improved the collision avoidance ratio with respect to MBCAP-i, e.g., from 88.04% to 98.22%, for the worst-case experiment (100 UAVs);
- **Hard collisions (d<4m)**. Represents the same metric, but with a more realistic threshold. In this case, we only considered that a collision had happened if the UAVs were closer than 4 m. As expected, the success ratio was higher (98.92%), but this was only detected in experiments with 100 UAVs, as in other cases the number of collisions was too low to compare;
- **Deadlocks avoided**. Represents the number of situations where an UAV surpassed the global timeout when waiting for other UAVs to solve another collision risk situation, but resumed its mission (transitions *f*, *i*, *j*, or *k* in Figure 2). Somehow the protocol had failed and the UAV waited an excessive amount of time because it was trying to solve a collision with an UAV that had already moved out of the contending area; in this situation, the protocol was able to detect that the UAV was no longer present and that the risk had gone, allowing the waiting UAV to go on with its mission. With MBCAP-e, the UAVs in this situation were only in extremely rare cases, i.e., 0.07% for 100 UAVs.
- **Deadlock failures**. Represents the number of situations where an UAV surpassed the global timeout while waiting for other UAVs to solve another collision risk situation, as it was not safe for it to go on with the mission (transition *g* in Figure 2). If a low-priority UAV was blocked in a state for too long and the high-priority UAV was already present in the conflict area, it should not continue with the mission, because resuming it could cause a collision. MBCAP-i showed an undesirable behavior, failing in many encounters, while with MBCAP-e no UAV needed to land.

To gain further insight on the protocol performance, we analyzed in detail the few collisions detected, finding that the collision risks between two UAVs were always avoided, meaning that collisions always happened when three or more UAVs met in the same area, and at the same time; in particular, problems only occurred when a third UAV stopped in the path that the high-priority UAV was following while overtaking the low-priority UAV. We considered this case a possible but improbable situation.

Regarding the behavior of the low-priority UAV, when it stops it can stand still while being overtaken, or it could move aside to allow the other UAV to go on. With MBCAP-i, it needed to move aside in 22.8% of the cases, while with MBCAP-e this occurred for 28.3% of the cases. This is due to the Waypointbehavior improvement included in the collision avoidance strategy (see Section 4.5 and Figure 10), as it forced the low-priority UAV to move further away from the path the high-priority UAV had to follow.

**Figure 18:**
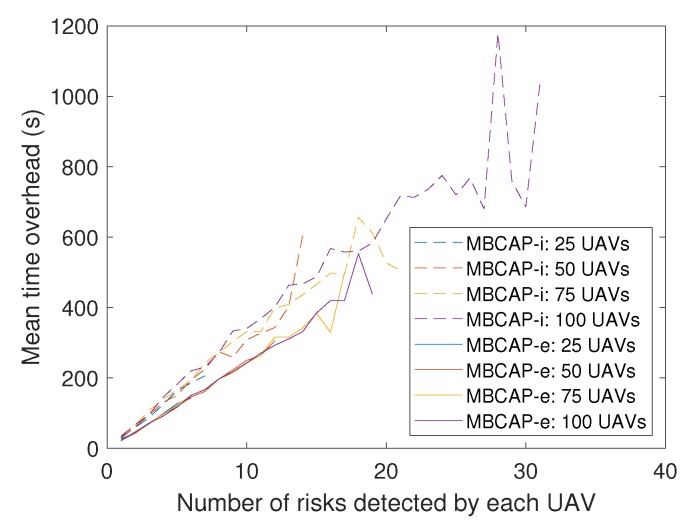
MBCAP-i vs. MBCAP-e: Global time overhead given the risks detected by each UAV.

**Figure 19:**
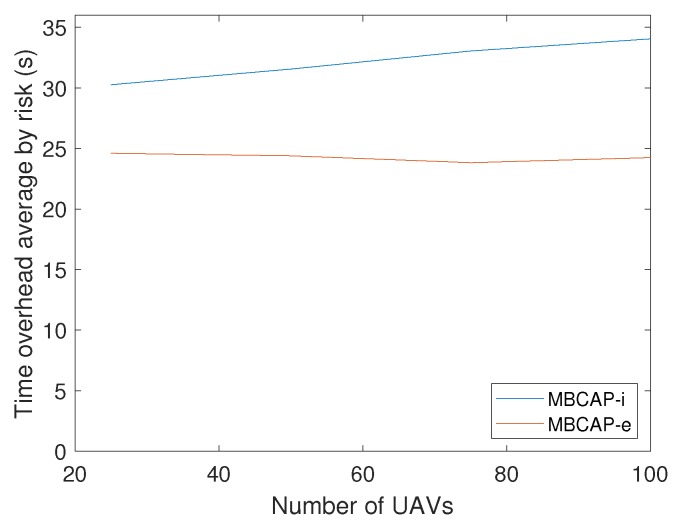
MBCAP-i vs. MBCAP-e: Time overhead by risk detected vs. number of UAVs.

Up to this point, we have compared the success ratio of MBCAP-i and MBCAP-e. Now we analyze the flight time overhead of both versions of the protocol. Table 5 shows the mean flight time and path length for an UAV using MBCAP-i, MBCAP-e, and without using the protocol. We can observe that, with MBCAP-e, an UAV needs to travel for an additional 35 meters on average, and consumes a mean extra time of 158 seconds at avoiding collisions, while with MBCAP-i it needs 51 extra meters and 306 extra seconds, respectively. The mean speed during the flight was also higher with MBCAP-e, showing that MBCAP-e was significantly more efficient at avoiding collisions than the previous version. Figure 18 shows the mean time overhead for an UAV during the whole experiment, given the number of times that UAV needed to stop to avoid a collision. We can observe that the slope of the line is nearly constant, independently of the number of UAVs included in the experiment. Also, we find that its slope is lower with MBCAP-e, as the UAV requires less time to avoid each collision. We also find that, the more UAVs present in the same experiment, the more collision risks per UAV were detected, and that with MBCAP-i an UAV detected more collision risks than with MBCAP-e. Finally, Figure 19 shows the mean time needed for an UAV to avoid a single collision depending on the number of UAVs flying around in the same experiment. It is clear that, with MBCAP-e, UAVs required less time to avoid a collision, with a mean value lower than 25 s, while with the previous version it needed more than 30 s.

Overall, experiments have shown that MBCAP-e added a significant improvement over the earlier version of this protocol when performing large scale simulations in a congested airspace. Furthermore, we found that the time overhead introduced by the protocol remained quite low (mean overhead of 25 s per risky situation solved).

## 6. Conclusions and Future Work

As new mission-based applications for multicopters emerge, the number of UAVs flying simultaneously also increases, and the risk of collision between them becomes higher. In addition, there are currently no collision avoidance protocols developed for UAVs from different owners when performing planned missions.

This work proposed MBCAP-e, an enhanced version of the MBCAP protocol that avoids collisions between multirotor UAVs performing planned missions by relying on a cooperative sense and avoid approach. Experimental results showed that MBCAP-e was able to avoid collisions between two UAVs in all cases, and with a success ratio of 98.22% in highly crowded environments (100 UAVs scenario). Experiments using real UAVs evidenced the resemblance between the simulated and real-life performance of MBCAP-e. In addition, we found the flight time overhead introduced by the protocol to be quite low and well bounded, considering the current lifespan of multicopter batteries. Overall, the effectiveness, reliability, and efficiency of MBCAP-e proved to be considerably higher when compared to its previous version (MBCAP-i). In fact, we found that collisions only took place with MBCAP-e when multiple UAVs were implied in the risky situation, and one of them remained stopped along the path of the high-priority UAV while taking over, a situation associated to the priority strategy adopted.

As future work, we plan to improve the priority strategy considering that different applications may need different priority levels, and so different alternatives for handling priority assignment will be studied. We will also study alternative collision avoidance strategies based on safety areas and roundabout-like manoeuvres [26] to further enhance the effectiveness of MBCAP-e at avoiding collisions, and to reduce the time overhead involved in collision avoidance manoeuvres.

## Figures and Tables

**Table 1:** MBCAP flight time overhead (min:s).

Scenario	UAV	MBCAP-i			MBCAP-e		
		on	off	Δt	on	off	Δt
1	1	3:41	3:03	0:38	3:29	2:59	0:30
	2	3:21	3:03	0:18	3:17	3:00	0:17
2	1	4:28	3:35	0:53	4:08	3:33	0:35
	2	3:32	3:03	0:29	3:23	2:59	0:24
3	1	3:59	3:03	0:56	3:41	2:59	0:42
	2	3:32	3:03	0:29	3:21	3:00	0:21
4	1	3:40	3:03	0:37	3:27	2:59	0:28
	2	3:21	3:03	0:18	3:16	3:01	0:15
5	1	3:51	3:03	0:48	3:34	2:59	0:35
	2	3:26	3:03	0:23	3:16	3:01	0:15
6	1	10:46	10:11	0:35	10:31	10:04	0:27
	2	30:47	30:27	0:20	30:33	30:17	0:16

**Table 2:** MBCAP flight time overhead (min:s) vs. wind.

Scenario	Wind	UAV	MBCAP-i	MBCAP-e		
			Δt	on	off	Δt
1	crosswind	1	0:38	3:29	3:00	0:29
	headwind	2	0:18	3:26	3:11	0:15
1	crosswind	1	0:38	3:30	3:01	0:29
	tailwind	2	0:18	3:15	2:58	0:17
2	headwind	1	0:53	4:09	3:34	0:35
	headwind	2	0:29	3:32	3:08	0:24
2	tailwind	1	0:53	4:07	3:32	0:35
	tailwind	2	0:29	3:22	2:58	0:24

**Table 3:** MBCAP-e flight time overhead (min:s). Simulation vs. real testbed.

Scenario	UAV	Simulation			Real testbed		
		on	off	Δt	on	off	Δt
1	1: quadcopter	3:53	3:25	0:28	3:56	3:21	0:35
	2: hexacopter	3:10	2:53	0:17	3:15	2:59	0:16
2	1: quadcopter	4:10	3:38	0:32	4:12	3:36	0:36
	2: hexacopter	3:57	3:33	0:24	4:08	3:43	0:25
3	1: quadcopter	3:39	2:56	0:43	3:39	2:56	0:43
	2: hexacopter	3:20	2:56	0:24	3:18	2:59	0:19
4	1: quadcopter	3:30	3:04	0:26	3:29	3:03	0:26
	2: hexacopter	3:20	3:02	0:18	3:29	3:03	0:26
5	1: quadcopter	3:31	2:54	0:37	3:49	2:55	0:54
	2: hexacopter	3:14	2:58	0:16	3:23	2:59	0:24

**Table 4:** MBCAP-i vs. MBCAP-e: Collision avoidance performance (mean value by experiment).

	MBCAP-i				MBCAP-e			
Number of UAVs	25	50	75	100	25	50	75	100
Collisions expected	6.5	16.5	45.5	84.25	6.5	16.5	45.5	84.25
Risks detected	28.17	132.92	338.75	659.42	23.08	105.08	249.08	438
Soft collisions (d<5 m)	0.58	1.83	3.42	10.08	0.08	0.08	0.58	1.5
Hard collisions (d<4 m)	0.58	1.67	2.58	8.42	0.08	0.08	0.58	1.08
Deadlocks avoided	0.08	1.67	4.33	10.83	0	0.33	0.25	0.58
Deadlock failures	0.33	3.42	8.75	21.42	0	0	0	0

**Table 5:** MBCAP-i vs. MBCAP-e: Performance comparison (mean value by experiment).

		Reference	MBCAP-i	MBCAP-e
Flight time (s)	Min.	3618	3691	3688
	Mean	3848	4154	4006
	Max.	4111	5511	4457
	Max. overhead	-	1120	553
Flight length (m)	Min.	34893	34916	34918
	Mean	36898	36949	36933
	Max.	39194	39258	39253
	Max. overhead	-	229	127
Mean speed (m/s)		9.59	8.9	9.22
